# Key role for Rac in the early transcriptional response to extracellular matrix stiffness and stiffness-dependent repression of ATF3

**DOI:** 10.1242/jcs.260636

**Published:** 2023-10-12

**Authors:** Irène Dang, Joseph A. Brazzo, Yongho Bae, Richard K. Assoian

**Affiliations:** ^1^ Department of Systems Pharmacology and Translational Therapeutics, University of Pennsylvania, Philadelphia, PA 19104, USA; ^2^ Department of Pathology and Anatomical Sciences, Jacobs School of Medicine and Biomedical Sciences, University at Buffalo, State University of New York, Buffalo, NY 14203, USA

**Keywords:** ECM, RNAseq, Cell cycling, Mechanotransduction, Proliferation, Rigidity

## Abstract

The Rho family GTPases Rac and Rho play critical roles in transmitting mechanical information contained within the extracellular matrix (ECM) to the cell. Rac and Rho have well-described roles in regulating stiffness-dependent actin remodeling, proliferation and motility. However, much less is known about the relative roles of these GTPases in stiffness-dependent transcription, particularly at the genome-wide level. Here, we selectively inhibited Rac and Rho in mouse embryonic fibroblasts cultured on deformable substrata and used RNA sequencing to elucidate and compare the contribution of these GTPases to the early transcriptional response to ECM stiffness. Surprisingly, we found that the stiffness-dependent activation of Rac was dominant over Rho in the initial transcriptional response to ECM stiffness. We also identified activating transcription factor 3 (ATF3) as a major target of stiffness- and Rac-mediated signaling and show that ATF3 repression by ECM stiffness helps to explain how the stiffness-dependent activation of Rac results in the induction of cyclin D1.

## Teaser

**Summary:** Rac plays a major role in the initial transcriptional response to extracellular matrix stiffness and mediates the stiffness-dependent repression of ATF3, resulting in the induction of cyclin D1.

## INTRODUCTION

Cell adhesion to the extracellular matrix (ECM) is essential for viability, motility and proliferation of many cell types. Adhesion to the ECM provides chemical information to the cell because many distinct ECM components bind to specific receptors on the cell surface to regulate intracellular signaling (Assoian and Schwartz, 2001; Boudreau and Jones, 1999; Larsen et al., 2006). Additionally, the ECM provides mechanical information to the cell, and the rigidity or stiffness of the ECM affects many of the signaling events and cellular fates originally attributed to cell adhesion (Discher et al., 2005; Janmey et al., 2015; Suresh, 2007). Cells can sense the stiffness of their microenvironment when ECM proteins such as fibrillar collagens bind to cell surface integrins (Bourgot et al., 2020; Lee and Juliano, 2004; Sun, 2021; Tang, 2020). Other ECM proteins such as fibronectin (FN) and vitronectin also bind to specific integrins and contribute to the information content of the cellular microenvironment.

The cytoplasmic domains of integrins lack intrinsic kinase activity, but they associate directly and indirectly with signaling molecules in dynamic macromolecular structures called focal complexes (FCs) and focal adhesions (FAs). FCs are located near the cell periphery and mature into FAs under force (Burridge and Guilluy, 2016; Burridge and Wittchen, 2013; Geiger and Bershadsky, 2001; Geiger and Yamada, 2011; Wolfenson et al., 2019). FAs contain many signaling molecules (Horton et al., 2016; Sastry and Burridge, 2000; Zaidel-Bar et al., 2007) and are also non-covalently linked to actin stress fibers, which mediate actomyosin contraction, generate intracellular tension and reinforce FA maturation and stabilization. ECM–integrin interactions with FCs and FAs ultimately control mechanotransduction to the nucleus and a number of stiffness-sensitive cell fates including motility, differentiation, proliferation and transformation (Assoian and Klein, 2008; Engler et al., 2006; Janmey et al., 2015; Paszek et al., 2005; Talwar et al., 2021; Wei et al., 2015; Yeung et al., 2005; Zajac and Discher, 2008).

The Rac and Rho GTPases are critical in mechanosensitive signaling and influence the cell fates described above (Burridge and Wennerberg, 2004; Hall, 2005; Lawson and Burridge, 2014; Pasapera et al., 2014; Provenzano and Keely, 2011; Ridley, 2015; Sahai and Marshall, 2003; Zohn et al., 1998). Rac activation leads to FC formation and cell spreading, whereas Rho activity directs stress fiber and FA formation and enforcement. Both Rac and Rho affect cell motility (Hall, 2005; Lawson and Burridge, 2014; Raftopoulou and Hall, 2004; Ridley, 2015; Sahai and Marshall, 2003). In contrast, we found that the activation of Rac, but not Rho, is required for the stimulatory effects of ECM stiffness on cyclin D1 and cell cycle progression from quiescent (G0) to S phase (Klein et al., 2009). In particular, the stiffness-dependent activation of FAK within FAs leads to the activation of p130Cas and eventually of DOCK180, a Rac guanine nucleotide exchange factor (GEF); the consequent stiffness-dependent activation of Rac and induction of lamellipodin is required for the mid-G1 phase expression of cyclin D1 (Bae et al., 2014; Brazzo et al., 2021). Among its many effects, cyclin D1 is perhaps best understood as an activator of cyclin-dependent kinases 4 and 6, which in turn, contribute to the release of E2Fs from the retinoblastoma-protein family and promotes S phase entry (Resnitzky and Reed, 1995; Sherr, 1993, 1995; Weinberg, 1995). Thus, the stiffness-dependent activation of Rac plays a key role in the stimulatory effect of ECM stiffness on cell cycling through the G1 phase. The third Rho family GTPase, Cdc42, is responsible for filopodia formation and directional motility (Cerione, 2004; Hall, 2005; Raftopoulou and Hall, 2004; Ridley, 2006), but its role in mechanosensitive cell fates is not as well understood.

In addition to their effects on cytoplasmic signaling, both Rac and Rho regulate transcription. The transcriptional effect is best understood for Rho, as Rho-dependent actin polymerization leads to nuclear translocation of MAL proteins [also known as myocardin-related transcription factors A (MRTFA) and B (MRTFB), or megakaryoblastic leukemia-1 and -2, respectively], co-activators of SRF genes (Miralles et al., 2003; Parmacek, 2007; Pipes et al., 2006; Sotiropoulos et al., 1999; Zhao et al., 2007). Additionally, both Rho and Rac affect nuclear translocation of the YAP/Taz transcriptional co-activators (Aragona et al., 2013; Dupont et al., 2011; Jang et al., 2017; Talwar et al., 2021). Nevertheless, much remains unknown about the relative contributions of Rac and Rho in the transcriptional response to ECM stiffness, particularly at the genome-wide scale. To address this gap in understanding, we used next-generation sequencing to determine and compare the effects of Rac versus Rho on the early transcriptional response to ECM stiffness.

## RESULTS AND DISCUSSION

### Specific inhibition of Rac and Rho activation during the early stages of cell adhesion and spreading

The development of the pharmacological inhibitors EHT1864 for Rac and CT04 for Rho represents a significant advance in addressing the effects of Rac versus Rho on cell function and circumvents major limitations of other analytical approaches. For example, dominant-negative constructs (Rac^N17^ and Rho^N19^, respectively) sequester GEFs that can affect both Rac and Rho GTPases, and, in some cases, Cdc42 as well (Cook et al., 2014; Debreceni et al., 2004). The well-established pharmacological Rac-GEF inhibitor NSC23766 does not inhibit all Rac GEFs (Shutes et al., 2007) and can inhibit Rho family GTPases beyond Rac (Levay et al., 2013). Genetic approaches are confounded by the presence of multiple Rac and Rho isoforms, with Rac1–3 and RhoA–C being the canonical family members (Cook et al., 2014). Therefore, knockout and/or RNAi-mediated depletion of multiple genes would be needed for each GTPase to ensure the absence of compensatory effects. Moreover, chronic approaches, such as those used for genetic depletion, also have the potential to confound the interpretation of results due to Rho family GTPase cross-regulation (Burridge and Wennerberg, 2004; Hall, 1998; Li et al., 2002; Sander et al., 1999).

EHT1864 directly binds to and specifically inhibits Rac1–3 by promoting the loss of bound nucleotide and inhibiting guanine nucleotide and GEF binding (Shutes et al., 2007). It thereby locks Rac into an inert state incapable of engaging downstream effectors (Shutes et al., 2007). CT04 is a membrane-permeable form of C3 transferase that specifically inhibits the Rho subfamily through ADP ribosylation (Narumiya and Thumkeo, 2018; Narumiya et al., 1988). The potential for long-term cross-regulation among the Rho family GTPases remains with use of these reagents, but this potential complication can be controlled experimentally because the drugs act quickly and can therefore be used with short incubation times.

**Fig. 1.**
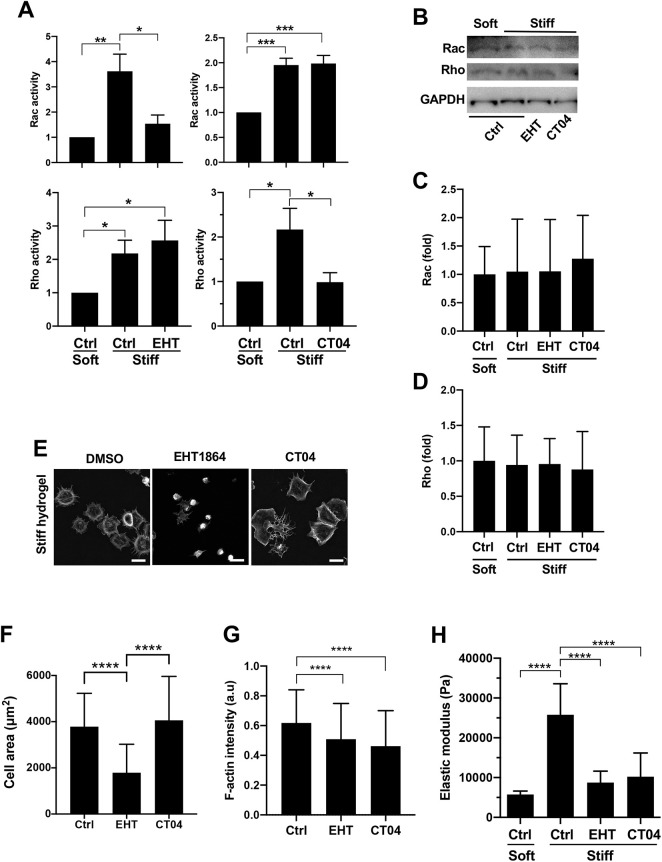
**Rac and Rho inhibition similarly reduce F-actin staining and cell stiffness.** Serum-starved MEFs pre-incubated with DMSO (Ctrl), EHT1864 or CT04 were plated on FN-coated hydrogels with 10% FBS for 1 h. (A) Rac and Rho activities were measured by G-LISA and plotted relative to the activity on the soft hydrogels (*n*=4). Graphs show mean+s.e.m. (B) Protein levels of Rac and Rho after 1 h treatment with EHT1864 or CT04 as described for panel A. GAPDH was used as the loading control. (C,D) Quantification of results in B. The graphs show mean+s.d. with results normalized to GAPDH abundance and plotted relative to the normalized signal on the soft hydrogels (*n*=4). (E) Maximum-projection images of phalloidin-stained MEFs. Scale bars: 50 µm. (F) Quantification of cell areas. The graphs show mean+s.d. *n*=259 (DMSO), 308 (EHT1864) and 222 (CT04) cells accrued from four (DMSO and CT04) or five (DMSO and EHT1864) independent experiments. (G) Phalloidin staining intensity normalized to cell area with results showing mean+s.d. *n*=137 (DMSO), 165 (EHT1864) and 152 (CT04) in cells accrued from three independent experiments. a.u., arbitrary units. (H) Cell stiffness as determined by AFM. The graph shows mean+s.e.m. (*n*=34 cells per condition accrued over four independent experiments). *P<0.05; **P<0.01; ***P<0.001; ****P<0.0001 [two-tailed unpaired *t*-tests, with the exception of testing for the inhibitory effects of EHT1864 and CT04 on their established targets (Rac and Rho, respectively) in panel A, where a one-tailed unpaired *t*-test was used].

To assess the utility of EHT1864 and CT04 in interrogating the early stiffness-sensitive transcriptome, we serum-starved mouse embryonic fibroblasts (MEFs), plated the cells on FN-coated polyacrylamide hydrogels and stimulated them with fetal bovine serum (FBS). This approach permits a synchronous exit from G0 and progression from G1 to S phase. We used a hydrogel with stiffness that mimics the stiffness found at sites of proliferation *in vivo* (20–25 kPa; denoted ‘stiff’) and promotes motility and S phase entry in cultured cells (Bae et al., 2014; Klein et al., 2009; Razinia et al., 2017; Shutova et al., 2017). We also used a hydrogel with a lower stiffness (4–6 kPa; denoted ‘soft’) that fails to stimulate proliferation and motility (Bae et al., 2014; Klein et al., 2009; Razinia et al., 2017; Shutova et al., 2017). Cells were cultured on hydrogels for 1 h before collection and analysis. Using this approach, we identified concentrations of EHT1864 and CT04 that inhibited Rac and Rho activity, respectively, without affecting the other GTPase (Fig. 1A) or Rac or Rho protein levels (Fig. 1B–D). Additionally, neither drug affected stiffness-stimulated Cdc42 activity (Fig. S1). Rac, but not Rho, inhibition reduced cell area (Fig. 1E,F), a consequence of impaired cell spreading (Movies 1, 2 and 3). F-actin abundance was mildly reduced in response to both EHT1864 and CT04 (Fig. 1D,G). Importantly, Rac and Rho inhibition similarly reduced intracellular stiffness as measured by atomic force microscopy (Fig. 1H).

### Rac is dominant over Rho in the early transcriptional response to ECM stiffness and mediates the stiffness-dependent repression of ATF3

**Fig. 2.**
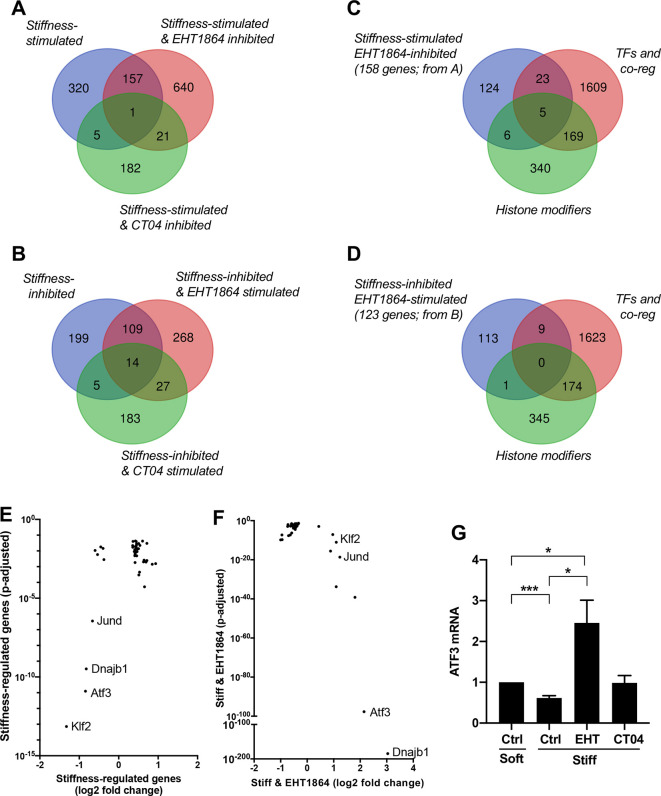
**Rac is dominant over Rho in the initial transcriptome-wide response to ECM stiffness and preferentially represses ATF3.** (A,B) Venn diagrams of differentially expressed genes in MEFs cultured with 10% FBS for 1 h on stiff versus soft hydrogels, stiff hydrogels with or without EHT1864, or stiff hydrogels with or without CT04. (C,D) The genes regulated by ECM and Rac in A,B were compared to GO gene lists for transcription factors (TFs), transcription co-regulators (co-reg) and histone modifiers. (E,F) Log_2_(fold change) values and adjusted *P*-values of the genes regulated by ECM stiffness and Rac and contained within the indicated GO terms above. (G) Serum-starved MEFs were plated on soft or stiff FN-coated hydrogels with 10% FBS for 1 h with DMSO (Ctrl), EHT1864 or CT04. *Atf3* mRNA levels were quantified by RT-qPCR. The graph shows mean+s.e.m. (*n*=4) with results normalized to the expression level on soft hydrogels. *P<0.05; ***P<0.001 (two-tailed unpaired *t*-tests).

Using the conditions identified in Fig. 1, we performed RNA sequencing (RNAseq) to interrogate the relative roles of Rac and Rho in transducing the early transcriptional response to ECM stiffness. The results showed that of 483 genes stimulated by ECM stiffness, one-third (158 genes) were inhibited by EHT1864, but only 1% (six genes) were inhibited by CT04 (Fig. 2A). Similarly, Rac was dominant over Rho when examining the genes that were inhibited by ECM stiffness (Fig. 2B): about 40% of stiffness-inhibited genes showed stimulated expression in response to EHT1864 (123 of 327), but only 6% showed stimulated expression in response to CT04 (19 of 327). Very few stiffness-regulated genes were affected by both the Rac and Rho inhibitors (Fig. 2A,B). Fisher’s exact tests showed that the overlap between the stiffness-regulated and EHT1864-regulated genes was significant (*P*<0.0001 and 0.019 for the stiffness-stimulated and -inhibited genes, respectively), whereas the overlap between stiffness-regulated genes and the few CT04-regulated genes was not significant (*P*=0.2 and 0.31 for stiffness-stimulated and -inhibited genes, respectively).

Several studies have identified a role for Rho in mechanosensitive transcription, most commonly through regulation of MAL or YAP/Taz translocation (see Introduction). The absence of Rho signals in our RNAseq data set does not question these findings but rather emphasizes that the initial global transcriptional response to ECM stiffness is much more dependent on Rac than Rho. The relative contributions of Rac and Rho to mechanosensitive transcription are likely to be time dependent, and the transcriptional contribution of Rho might increase as cells attach, spread and develop force in response to stiff substrata.

In an effort to better understand this transcriptome-wide response, we searched for transcriptional regulators within the set of genes that were inversely regulated by ECM stiffness and Rac. The 158 genes that were stimulated by ECM stiffness and inhibited by EHT1864 (Fig. 2A) and the 123 genes that were inhibited by ECM stiffness and stimulated by EHT1864 (Fig. 2B) were compared to the Gene Ontology (GO) lists of transcription factors, transcriptional co-regulators and histone modifiers (Fig. 2C,D). The lists of overlapping genes in Fig. 2C,D (Table S1) were combined and graphed by fold-change values and adjusted *P*-values to visualize the relative robustness of their responses to ECM stiffness and Rac inhibition with EHT1864 (Fig. 2E,F). The results showed that, as a group, neither transcriptional co-regulators nor histone modifiers were strongly regulated during the initial transcriptome-wide response to ECM stiffness and Rac. However, the mRNAs for two transcription factors (Klf2 and ATF3) were repressed by ECM stiffness with high statistical significance (Fig. 2E). Of those two, the repression of *Atf3* mRNA was much more robustly reversed by inhibition of Rac activity with EHT1864 (Fig. 2F). Note that the data shown in Fig. 2E,F also revealed a strong effect of ECM stiffness and Rac inhibition on the expression level of *Dnajb1* mRNA. Though associated with fibrolamellar carcinoma as a fusion protein (Dinh et al., 2022), little is known about Dnajb1 biology, and we therefore decided to focus our studies on ATF3. See Table S1 for gene lists corresponding to Fig. 2A–F. Real-time quantitative PCR (RT-qPCR) confirmed that *Atf3* mRNA levels were downregulated by culturing MEFs on a stiff substratum and upregulated by treatment with EHT1864. We note that the RNAseq detected a small effect of CT04 on *Atf3* mRNA expression compared to that of EHT1864 (Table S1), but a direct analysis of isolated mRNAs by RT-qPCR confirmed that the effect of CT04 on *Atf3* mRNA levels in cells on stiff hydrogels was small (Fig. 2G). Immunoblotting showed that the impacts of ECM stiffness, EHT1864 and CT04 on *Atf3* mRNA were similar to those on the protein (Fig. S2).

### Stiffness- and Rac-mediated repression of ATF3 is linked to the expression of cyclin D1

**Fig. 3.**
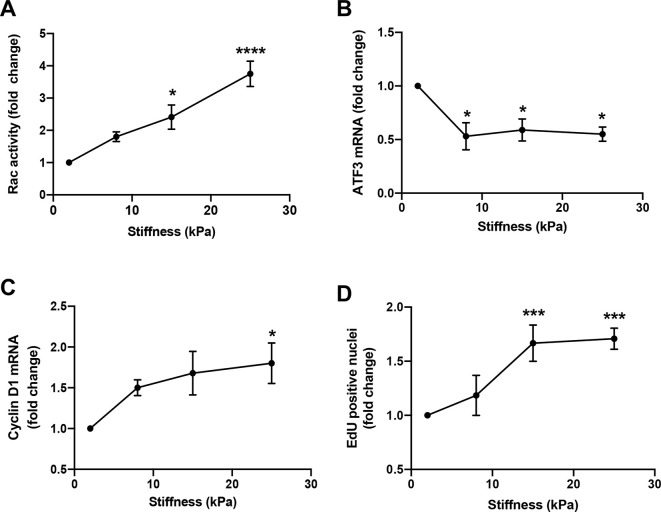
**Dose-dependent effects of ECM stiffness on Rac–GTP, *Atf3* mRNA, cyclin D1 mRNA and S phase entry.** Serum-starved MEFs were incubated in DMEM containing 10% FBS on FN-coated hydrogels of increasing stiffness (∼2, 8, 15 and 25 kPa). (A) Rac–GTP levels determined at 1 h and graphed relative to Rac activity on the softest hydrogel. Results show mean±s.e.m. (*n*=4). (B,C) *Atf3* and cyclin D1 mRNA levels determined after 9 h and graphed relative to the mRNA levels on the softest hydrogel. Results show mean±s.e.m. (*n*=3). (D) The percentage of EdU-positive nuclei determined at 24 h and graphed relative to EdU incorporation on the softest hydrogel. Results show mean±s.d. (*n*=3). Statistical significance for each panel was determined by one-way ANOVA; asterisks show the results of Dunnett's post-tests relative to the softest hydrogel. *P<0.05; ***P<0.001; ****P<0.0001.

ATF3 belongs to the ATF/CREB family of transcription factors, has pleiotropic effects on cells, and is canonically induced in response to various stress signals (Fan et al., 2002; Hai and Hartman, 2001; Hai et al., 1989; Ku and Cheng, 2020; Rohini et al., 2018). Intriguingly, ATF3 has also been described as a repressor of cyclin D1 transcription, with direct binding to an inhibitory ATF3 site in the cyclin D1 promoter (James et al., 2006; Lu et al., 2006). As indicated above, cyclin D1 is a key regulator of progression through the G1 phase and a major target of ECM stiffness-mediated signaling (Bae et al., 2014; Klein et al., 2009). By plating FBS-stimulated MEFs on FN-coated hydrogels of increasing stiffness, we found that the levels of *Atf3* (Fig. 3B) and cyclin D1 (*Ccnd1*) (Fig. 3C) mRNAs and S phase entry (Fig. 3D) correlated with the level of Rac activity (Fig. 3A). In agreement with Fig. 2, the correlation to Rac activity was inverse for *Atf3* mRNA but direct for cyclin D1 mRNA and S phase entry. ATF3 and cyclin D1 protein levels showed the same inverse pattern as was seen for their mRNAs (Fig. S3).

**Fig. 4.**
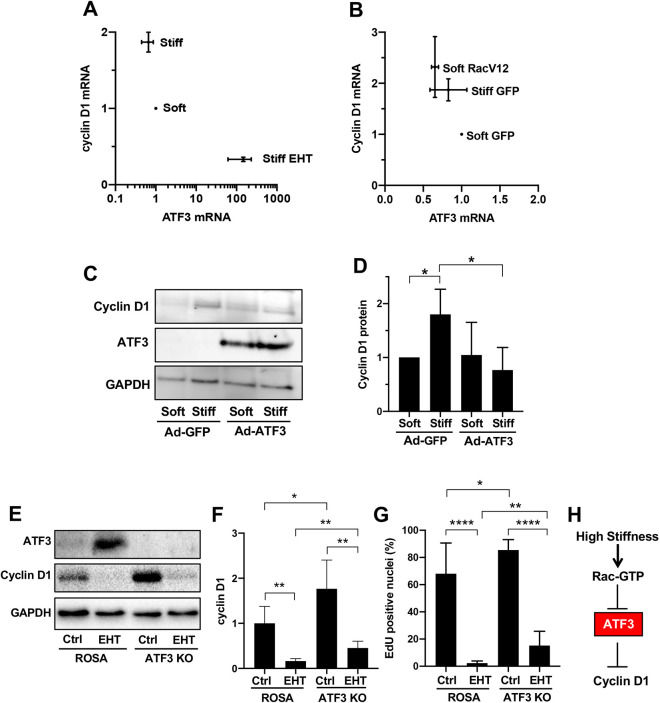
**ATF3 repression linked to stiffness-dependent cyclin D1 expression.** (A) Serum-starved MEFs on soft or stiff FN-coated hydrogels in DMEM containing 10% FBS were treated with vehicle (DMSO) or EHT1864 for 9 h. *Atf3* and cyclin D1 mRNA levels were determined from the same lysates and normalized to mRNA expression levels in cells on soft hydrogels. Results show mean±s.e.m. (*n*=3). (B) MEFs were infected with adenoviruses encoding GFP (control) or Rac^V12^, serum-starved, and cultured and analyzed as in panel A. Results show mean±s.e.m. (*n*=3). (C) MEFs infected with adenoviruses (Ad) encoding GFP (control) or ATF3 were serum-starved, incubated on FN-coated hydrogels with 10% FBS for 15 h, and analyzed by immunoblotting for cyclin D1 and ATF3 with GAPDH as the loading control. (D) Quantification of the immunoblot results in C. The graph shows mean+s.d. with results normalized to GAPDH abundance and plotted relative to the normalized cyclin D1 signal on the soft hydrogels (*n*=3). (E,F) Serum-starved ROSA and ATF3 KO MEFs were incubated on stiff FN-coated hydrogels with DMSO (Ctrl) or EHT1864 for 9 h. Lysates were analyzed for the levels of ATF3 and cyclin D1 by immunoblotting. GAPDH was used as the loading control. Panel E shows results from ROSA clone R12 and ATF3 KO clone 1-20, and panel F shows quantification of the combined results from ROSA clones R11, R12 and R15 and ATF3 KO clones 1-20 and 1-29. Data were accrued from four independent experiments, and the graph shows mean+s.d. with results normalized to GAPDH abundance and plotted relative to the normalized cyclin D1 signal in the ROSA control. (G) S phase entry was analyzed by EdU incorporation in ROSA clones (R3, R11, R12 and R15) and ATF3 KO clones (1-20, 1-29, 1-44, 1-48 and 1-49) after serum starvation and incubation on stiff FN-coated hydrogels in DMEM containing 10% FBS for 24 h with DMSO (Ctrl) or EHT1864. Results show mean+s.d. *n*=7 for the ROSA clones and *n*=8 for the ATF3 KO clones. (H) Model showing that cyclin D1 is regulated by ECM stiffness and Rac through ATF3. *P<0.05; **P<0.01; ****P<0.0001 (D, two-tailed unpaired *t*-tests; F and G, one-tailed unpaired *t*-tests).

We then compared the effect of Rac inhibition and activation on the expression of *Atf3* and cyclin D1 mRNAs within the same lysates. RT-qPCR revealed an inverse correlation between the expression of *Atf3* and cyclin D1 mRNAs in response to ECM stiffening (Fig. 4A; compare ‘Soft’ versus ‘Stiff’). Moreover, both the stiffness-dependent repression of *Atf3* mRNA and induction of cyclin D1 mRNA were reversed by Rac inhibition with EHT1864 (Fig. 4A; compare ‘Stiff’ with ‘Stiff EHT’). Conversely, enforced Rac activity (by ectopic expression of Rac^V12^) in MEFs on soft hydrogels strongly decreased the levels of *Atf3* mRNA, whereas cyclin D1 mRNA levels were increased (Fig. 4B). This inverse relationship between stiffness-dependent ATF3 repression and cyclin D1 induction is causal because forced expression of ATF3 in cells on stiff hydrogels was sufficient to block the stiffness-dependent induction of cyclin D1 (Fig. 4C,D).

The effect of Rac activity on cell spreading is related to Arp2/3, a master regulator of cortical actin branching and a canonical Rac target (Goley and Welch, 2006). Pharmacological inhibition of Arp2/3 with CK666 phenocopied the effect of Rac inhibition with EHT1864, overriding the effect of ECM stiffness on *Atf3* mRNA downregulation and cyclin D1 mRNA upregulation; its inactive analog, CK689, did not have an effect (Fig. S4). These data extend early studies showing that polymerized actin and cell spreading are required for cyclin D1 upregulation (Böhmer et al., 1996; Huang et al., 1998), and more recent work demonstrating that actin assembly and, particularly, cortical branched actin promotes cell cycle progression and proliferation (Mohan et al., 2019; Molinie et al., 2019). Collectively, these results indicate that the effects of Rac on the expression of *Atf3* and cyclin D1 mRNAs are closely related to the effects of Rac on cell spreading and actin organization. Additionally, these CK666 data provide independent support for our use of EHT1864 to assess Rac function on the early transcriptome-wide response to ECM stiffness.

Given that ATF3 expression reduced cyclin D1 levels, we used CRISPR-Cas9 methodology to delete *Atf3* from MEFs and determine if cyclin D1 levels and S phase entry were increased. We generated several *Atf3*-deficient clones [hereafter called ATF3 knockout (KO) cells] as well as several control clones in which MEFs were transfected with *ROSA26* (hereafter ROSA) rather than *Atf3* gRNA (Fig. S5A; Table S2). Phenotypically, the lack of ATF3 did not have an obvious morphologic effect on the cells, nor did we detect large changes in cell area or F-actin intensity in the ROSA control versus that in ATF3 KO MEFs incubated on stiff FN-coated hydrogels for 1 or 9 h (Fig. S5B–D). Cyclin D1 localization was mostly nuclear in both the control and ATF3 KO MEFs (Fig. S6A). However, serum-stimulated ATF3 KO MEFs on stiff hydrogels displayed an increase in cyclin D1 expression relative to that in the ROSA controls (Fig. 4E,F; Fig. S6B). Rac inhibition with EHT1864 reduced the abundance of cyclin D1 (Fig. 4E,F) in both the ROSA control and ATF KO MEFs (compare bar 1 to bar 2 and bar 3 to bar 4 in Fig. 4F), indicating – not surprisingly – that Rac activity drives cyclin D1 expression and S phase entry by pathways other than ATF3. Nevertheless, inhibition of cyclin D1 expression by EHT1864 treatment was greater in the ROSA controls than in the ATF3 KO cells (mean inhibitions were >6-fold versus <4-fold, respectively, in Fig. 4F). The cyclin D1 promoter is very complex, and it is not thought that one or even a few transcription factors dominate the induction of cyclin D1 mRNA in response to mitogenic stimulation (Klein and Assoian, 2008). Thus, our results are consistent with ATF3 having a contributory effect in the stiffness-dependent expression of cyclin D1.

The increased expression of cyclin D1 seen in ATF3 KO cells was also associated with an increase in S phase entry (Fig. 4G; compare bars 1 and 3). As with cyclin D1, EHT1864 inhibited S phase entry in both the ROSA control and ATF3 KO cells (compare bar 1 to bar 2 and bar 3 to bar 4 in Fig. 4G), but the ATF3 KO effect on S phase entry was stronger than its effect on cyclin D1: mean inhibition of 5-ethynyl-2′-deoxyuridine (EdU) incorporation by EHT1864 was almost 30-fold in the ROSA controls and <6-fold in the ATF3 KO MEFs. These data suggest that repression of ATF3 might regulate stiffness-dependent G1-phase cell cycle events beyond cyclin D1. Indeed, others have reported a repressive effect of ATF3 on cyclin A, cyclin E and Cdk2 levels, and ectopic expression of ATF3 suppresses cell cycle progression from G1 to S phase (Fan et al., 2002; James et al., 2006; Lu et al., 2006). We also acknowledge reports suggesting that ATF3 promotes cell proliferation (Allan et al., 2001; Perez et al., 2001). Use of different cell types and experimental conditions likely affect the cellular response to alterations in ATF3 levels. Our use of substrata of physiologically relevant stiffness likely provides additional insight not attainable with cells cultured on rigid (glass or plastic) surfaces.

ATF3 can act as a transcriptional activator or repressor (Fan et al., 2002; Hai and Hartman, 2001; Hai et al., 1989; Ku and Cheng, 2020; Rohini et al., 2018). ATF3 homodimers can inhibit gene targets directly through an association with histone deacetylase 1, whereas ATF3-containing heterodimers can either positively or negatively regulate gene expression. Whether ECM stiffness and Rac control the positive and negative cell cycle effects of ATF3 through homo- or hetero-dimerization and how this might contribute to cyclin D1 gene expression, S phase entry and nuclear mechanosensing (Dahl et al., 2008; Kirby and Lammerding, 2018; Swift and Discher, 2014; Wang et al., 2009) are important but complex matters for further study.

Increased tissue stiffness is seen in many pathological microenvironments, such as breast and pancreatic tumors, lung and liver fibrosis, and cardiovascular disease (Duprez and Cohn, 2007; Gehler et al., 2013; Keely, 2011; Kothapalli et al., 2012; Levental et al., 2009; Liu et al., 2010; Maneshi et al., 2021; Paszek et al., 2005; Piersma et al., 2020; Tung et al., 2015; Zhang et al., 2021), and has even been considered as a prognostic factor in cancer progression (Reid et al., 2017; Wei and Yang, 2016). Our data indicate that ATF3 is an early transcriptional target of increased ECM stiffness. Taken together with our previous work (Bae et al., 2014; Brazzo et al., 2021; Klein et al., 2009), they also provide the framework of a complete mechanosensitive signaling pathway beginning outside the cell (with the ECM), extending through integrins, FAs and Rac in the cytoplasm, and ending with reduced ATF3 expression and transcriptional upregulation of cyclin D1 in the nucleus (Fig. 4H). As ECM stiffening is a hallmark of several diseases and as ATF3 has widespread effects on cells, its downregulation in pathologically stiff microenvironments might be an early event that amplifies transcriptional misregulation in multiple mechanosensitive processes and pathologies.

## MATERIALS AND METHODS

### Cell culture, pharmacological inhibition, ectopic expression and GTPase activity assays

MEFs were generated from embryonic (E) day 12.5–14.5 C57BL/6J mouse embryos, spontaneously immortalized using standard procedures, and cultured in Dulbecco's modified Eagle medium (DMEM; 10-014-CV, Corning) containing 10% FBS (F2442, Sigma-Aldrich) at 37°C with 10% CO_2_. All work with mice was reviewed and approved by the University of Pennsylvania institutional animal care and use committee. Our early studies showed that the ECM and actin cytoskeleton are required for cell cycling from G0 to S phase, until the mid-G1 phase phosphorylation of Rb (Böhmer et al., 1996). To examine the global response of MEFs to the ECM, Rac and Rho in this portion of the cell cycle, we serum-starved the cells (typically for 48 h) and then incubated them with 10% FBS for times from 1 to 24 h. For serum starvation, near-confluent monolayers were incubated for 48 h with DMEM containing 1 mg/ml heat-inactivated fatty-acid free bovine serum albumin (BSA). To regulate ECM stiffness, the cells were cultured on polyacrylamide hydrogels coated with 5 µg/ml FN (341631, EMD) (Cretu et al., 2010; Klein et al., 2007) with 12-, 25- or 40-mm round coverslips, as well as 24×40-mm rectangular coverslips. Hydrogel stiffness ranged from 2–4 kPa (soft) to 20–25 kPa (stiff). The coverslips were collected, and the cells were fixed for immunofluorescence (12-mm coverslips) or processed for RNA (25-mm coverslips) or total protein (40-mm or 24×40-mm coverslips) extraction.

#### Acute inhibition of Rac or Rho

MEFs were cultured in DMEM containing 10% FBS to 70–80% confluency and then serum-starved in DMEM containing 1 mg/ml BSA. After 48 h, the cells were trypsinized, resuspended in DMEM containing 1 mg/ml BSA and incubated in suspension for 30 min at 37°C in 10% CO_2_ and 10 µM EHT1864 (3872, Tocris or 17258, Cayman Chemical) or 2 µg/ml CT04 (CT04-A, Cytoskeleton). Stock solutions of EHT1864 and CT04 were generated as per the manufacturers’ instructions. When experiments included EHT1864, the vehicle control contained the corresponding dilution of DMSO. These pretreated cells were collected by gentle centrifugation, resuspended in DMEM containing 10% FBS, and plated on FN-coated hydrogels in the continued presence of inhibitor.

#### Ectopic expression

MEFs were cultured at 70–80% confluency and infected with adenoviruses encoding Rac^V12^ (a kind gift of Chris Chen, Boston University), Ad-GFP-h-ATF3 (Vector Biolabs) or GFP, largely as described previously (Bae et al., 2014; Klein et al., 2007). The adenovirus dilutions used resulted in ∼80% cell infection as judged by the GFP signal. The infected cells were washed with DMEM containing 1 mg/ml BSA, serum-starved, trypsinized and plated on FN-coated polyacrylamide hydrogels as described above for 9 h prior to analysis by RT-qPCR and immunoblotting.

#### Rho family GTPase activity assay

Cells treated with EHT1864 and CT04 as described above were collected, and Rac or Rho activity was assessed in duplicate using the G-LISA small G-protein activation assay kit (BK128, BK124, Cytoskeleton) according to the manufacturer's instructions. Briefly, serum-starved MEFs were replated on soft and stiff FN-coated hydrogels and stimulated with 10% FBS for 1 h. Total cell lysates were prepared with the provided lysis buffer (pre-chilled on ice), and the protein concentration was measured by Coomassie binding (Bio-Rad). Equal amounts of protein were added to each well of a G-LISA plate and incubated for 30 min at 4°C. Bound Rac was detected using the provided anti-Rac primary and HRP-tagged secondary antibodies as per the manufacturer's instructions. Finally, HRP detection reagents were added and the resulting colorimetric reaction was quantified by measuring absorbance at 490 nm in a microplate spectrophotometer.

### Atomic force microscopy

Intracellular stiffness was measured by plating cells for 1 h on 18-mm soft or stiff FN-coated polyacrylamide hydrogels with 10% FBS. The intracellular stiffness of single adherent cells was measured using a DAFM-2X Bioscope (Veeco) mounted on an Axiovert 100 microscope (Zeiss) in contact mode. Cells were indented against a standard silicon nitride cantilever (spring constant=0.06 N/m) with a conical tip (40 nm in diameter). The elastic modulus (stiffness) was calculated by fitting the first 600 nm of tip deflection from the horizontal with the Hertz model for a cone. The tip was placed near the edge of the cell to measure intracellular stiffness. Several atomic force microscopy (AFM) measurements were taken of each cell (with seven to ten cells tested per condition and per experiment), and mean stiffness was calculated for each cell using a custom MATLAB script generously provided by Paul Janmey (University of Pennsylvania). Results from 34 cells per condition were accrued over four independent experiments and graphed.

### Fluorescence microscopy and quantification of F-actin intensity and cell area

Hydrogels were washed with PBS and the cells were fixed in 3.7% formaldehyde (15 min at room temperature), washed three times with PBS, permeabilized with 0.4% Triton X-100 in PBS for 10 min, washed once with PBS, and blocked in 2% BSA and 0.2% Triton X-100 (30 min at room temperature). Primary antibodies to ATF3 (1:100, sc-188, Santa Cruz Biotechnology) and cyclin D1 (1:100, sc-450, Santa Cruz Biotechnology) were diluted in 2% BSA and 0.2% Triton X-100 and incubated with the cells for 1 h. The cells were then washed three times in PBS containing 2%, BSA and 0.2% Triton X-100 and incubated with the secondary antibody (diluted 100-fold) for 1 h at room temperature. The immunostained cells were then washed twice, and the coverslips were mounted using DAPI fluoromount G (0100-20, SouthernBiotech). To quantify F-actin signal intensity, fixed cells that had been stained for 1 h with Alexa Fluor 594 Phalloidin (A12381, Thermo Fisher Scientific) in 2% BSA and 0.2% Triton X-100 were washed three times in PBS containing 0.2% Triton X-100 and mounted as described above. Quantification of cell area was performed using ImageJ: a threshold was set to cover the whole cell surface, holes were filled using the Process→Binary→Fill Holes command, and then the individual cell areas were measured (Dang et al., 2013). The phalloidin signal for each cell was measured, set on an arbitrary threshold based on the DMSO control cell intensity. F-actin intensity was then normalized to cell area for each cell.

### RNAseq and bioinformatic analysis

Quadruplicate samples were generated for MEFs cultured in four different conditions: (1) soft hydrogels, (2) stiff hydrogels, (3) stiff hydrogels with EHT1864 and (4) stiff hydrogels with CT04 as described above for ‘Acute Inhibition of Rac or Rho’. Total RNA was extracted using TRIzol reagent (Invitrogen), further purified with an RNeasy kit (74106, Qiagen), and prepared for RNA sequencing using TruSeq RNA Stranded mRNA (Illumina) and 100 base pair paired-end reads. Sequencing coverage was ∼40×10^6^ reads. Salmon (https://combine-lab.github.io/salmon/) was used to count the data against the transcriptome defined in Gencode vM28, which was built on the genome GRCm39. Several Bioconductor packages (https://bioconductor.org), as described below, were used for subsequent steps. The transcriptome count data were annotated and summarized to the gene level with tximeta and further annotated with biomaRt. A principal component analysis was performed with PCAtools, and this led to the exclusion of one of the four replicates of MEFs cultured on a soft hydrogel. Raw feature counts of the remaining 15 samples were normalized and analyzed for differential expression using DESeq2. Venn diagrams were generated from the DESeq2 output based on cut-offs of 0.32 log_2_ (approximately >1.25 log_10_) fold change (positive or negative), adjusted *P*-value <0.05, and >500 for base mean intensity. We identified the genes that were inversely regulated by ECM stiffness and Rac, or ECM stiffness and Rho, as defined as a change in sign of log_2_(fold change). We then compared those lists to GO gene lists for transcription factors, transcription co-regulators and histone modifiers (GO terms 0003700, 0003712, and 0016570, respectively). Primary data from the RNAseq have been deposited into the Gene Expression Omnibus database and can be found at GSE236266.

### CRISPR/Cas9-mediated deletion of *Atf3*

The CRISPR sgRNA to mouse *Atf3* (5′-CCAGCGCAGAGGACAUCCGA-3′) and ROSA26 (5′-GAACAUAAAUGGCAACAUCU-3′) were obtained from Synthego Corporation. The sgRNAs were diluted to 30 µM and Cas9 nuclease to 20 µM. MEFs were seeded in 12-well plates and grown to 70–80% confluency. Cells were transfected with ribonucleoprotein (RNP) complexes using a NEON electroporation system (Invitrogen) and an RNP complex ratio of 9:1. Cells were electroporated using one pulse at 1350 V for 30 ms and then incubated in DMEM containing 10% FBS for 2–3 days. For clonal expansion, each well was trypsinized and diluted such that an average of 0.5 cells were plated per well in 96-well plates. Cells were cultured until they reached ∼70% confluency and then subjected to Sanger sequencing. The ROSA26 primers were 5′-ACATTTGGTCCTGCTTGAACA-3′ (forward) and 5′-ACATTTGGTCCTGCTTGAACA-3′ (reverse). The *Atf3* primers (for gRNA-1) were 5′-GTAGGCTGTCAGACCCCATG-3′ (forward) and 5′-GGTGCACACTATACCTGCTC-3′ (reverse). Sanger sequencing data were uploaded to the ICE CRISPR Analysis Tool (Synthego) to assess the *Atf3* editing efficiency for each clone. Clones with a predicted knockout efficiency >95% were analyzed further.

### RT-qPCR

Total RNA was extracted and purified from MEFs using the Quick-RNA Miniprep Plus Kit (Zymo Research). cDNAs were prepared using equal amounts of total RNA diluted into TaqMan reverse transcription master mix (Thermo Fisher Scientific) and then processed according to the manufacturer's instructions. Gene expression was quantified by RT-qPCR performed in duplicate using TaqMan Universal PCR master mix, ∼20 ng of total RNA for reverse transcription, and 5 ng of the reverse transcription product in the qPCR. Taqman assays (Thermo Fisher Scientific) were used for *Atf3* (Mm00476033_m1) and *Ccnd1* (Mm00432359_m1.). The primer probe set for 18S rRNA has been described previously (Klein et al., 2007). The level of mRNA expression for each gene was determined by the ddCt method and plotted relative to 18S rRNA expression.

### Immunoblotting

MEFs on hydrogels were collected in RIPA buffer (25 mM Tris-HCl, pH 8, 150 mM NaCl, 1% NP-40, 0.5% deoxycholate and 0.1% SDS) containing protease inhibitors (5872S, Cell Signaling Technology). Cell lysates were centrifuged to remove nuclei and debris. Supernatants were collected and their protein concentrations were determined using a Bradford protein assay (5000006, Bio-Rad Laboratories). Equal amounts of proteins were diluted in LDS Sample Buffer (Invitrogen) with β-mercaptoethanol, and fractionated on 12% SurePage polyacrylamide gels (GenScript). In some experiments, cells were extracted in 5× SDS sample buffer with β-mercaptoethanol and fractionated on Tris-glycine polyacrylamide gels as described previously (Bae et al., 2014). Proteins were transferred onto nitrocellulose or polyvinylidene difluoride (PVDF) membranes. The membranes were saturated with 5% BSA in 1× TBS (20 mM Tris-HCl, pH 7.5, 150 mM NaCl) with 0.1% Tween-20 and probed with primary antibodies to ATF3 (1:200, sc-188, Santa Cruz Biotechnology or 1:500, NBP1-85816, Novus Biologicals), cyclin D1 (1:200, sc-20044, Santa Cruz Biotechnology or 1:500, 681902, BioLegend), Rac1/2/3 (1:100, 2465, Cell Signaling Technology), RhoA/B/C (1:200, MA1-011, Thermo Fisher Scientific) or GAPDH (loading control; 1:1000, MA5-15738, Thermo Fisher Scientific). The secondary antibodies used were ECL anti-rabbit HRP (1:500, 3144, GE Healthcare) and ECL anti-rabbit HRP (1:500, 3143, GE Healthcare). Antibodies were diluted in the same TBS buffer, and signals were detected by enhanced chemiluminescence with an ImageQuant LAS 4000. Blots were quantified with ImageJ and normalized to the corresponding loading control. See Fig. S7 for images of the uncropped gels used in the figures.

### Determination of S phase entry by EdU incorporation

Cells were starved in DMEM containing 1 mg/ml BSA for 48 h, trypsinized, and incubated for 24 h on hydrogels in DMEM containing 10% FBS and 10 µM EdU (Invitrogen). EdU was visualized using the Click-iT EdU Imaging Kit (Invitrogen) according to the manufacturer's instructions. Coverslips were mounted onto glass microscope slides using DAPI fluoromount G for manual counting of DAPI-stained and EdU-positive nuclei. We used these data to calculate the percentage of cells with EdU-positive nuclei as a measure of S phase entry.

### Statistical analysis

Statistical significance was determined using Prism (GraphPad) software. Graphs show means±s.d. unless the independent experiments generated means, in which case the error bars show s.e.m. Unless noted otherwise, unpaired *t*-tests were used to compare data with *P<0.05, **P<0.01, ***P<0.001 and ****P<0.0001. Outliers were removed based on Grubbs' test. The *t*-tests were two-tailed unless testing for an effect in a specific direction.

## Supplementary Material

10.1242/joces.260636_sup1 Supplementary information
