# Supplementary material for: Key role for Rac in the early transcriptional response to extracellular matrix stiffness and stiffness-dependent repression of ATF3
Source: J Cell Sci. 2023 Oct 12;136(19):jcs260636. doi: 10.1242/jcs.260636 (PMC10617619; doi:10.1242/jcs.260636)
Supplement: Supplementary information [file joces-136-260636-s1.pdf]

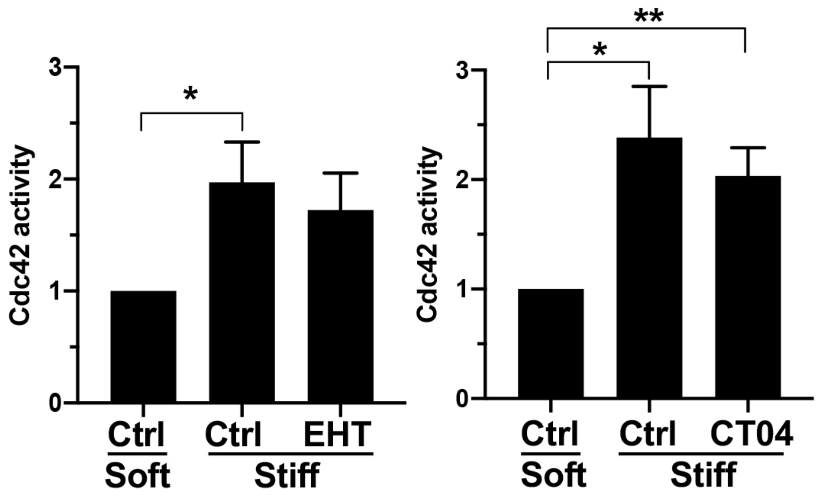

Fig. S1. Cdc42 activity is not affected by EHT1864 or CT04. Lysates from the experiments in Fig 1A (n=4) were analyzed for the levels of active Cdc42. See the legend to Fig. 1A for details. Statistical significance was determined by 2-tailed, unpaired t-tests.

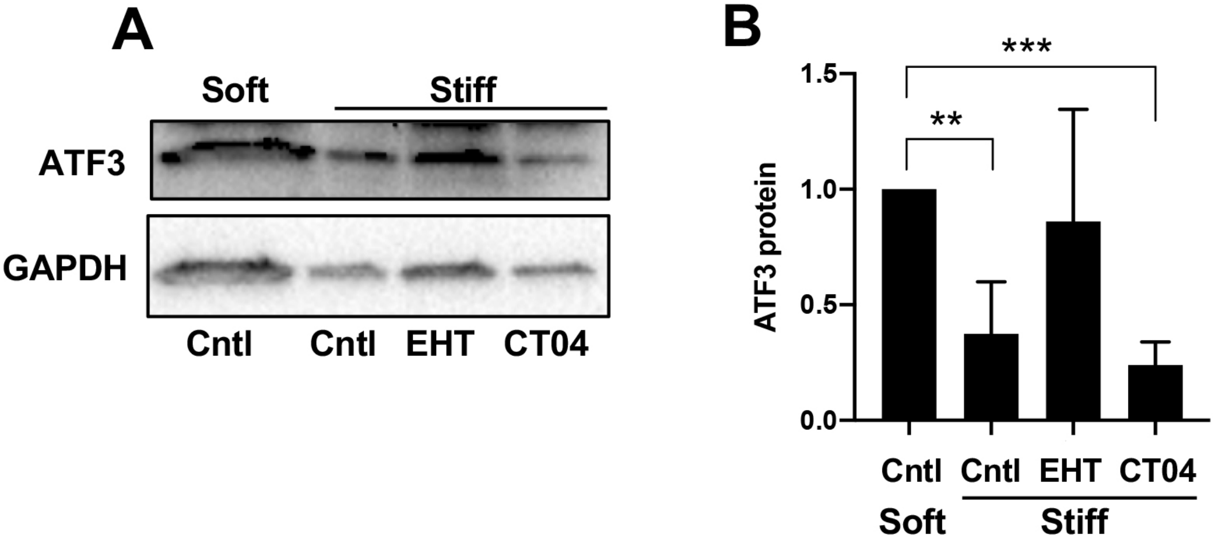

Fig. S2. Effects of ECM stiffness, EHT1864 and CT04 on ATF3 protein. Starved MEFs were plated on soft or stiff FN-coated hydrogels with 10% FBS and either DMSO (vehicle control; Cntl), EHT1864, or CT04 for 5 hr. **(A)** The cells were analyzed by immunoblot for ATF3 with GAPDH as the loading control. **(B)** Quantification of immunoblot results in A; the graph shows mean + SD with results normalized to GAPDH and plotted relative to the normalized ATF3 signal on the soft hydrogels; n=4-5. Statistical significance was determined by 2-tailed, paired t-tests.

Journal of Cell Science • Supplementary information

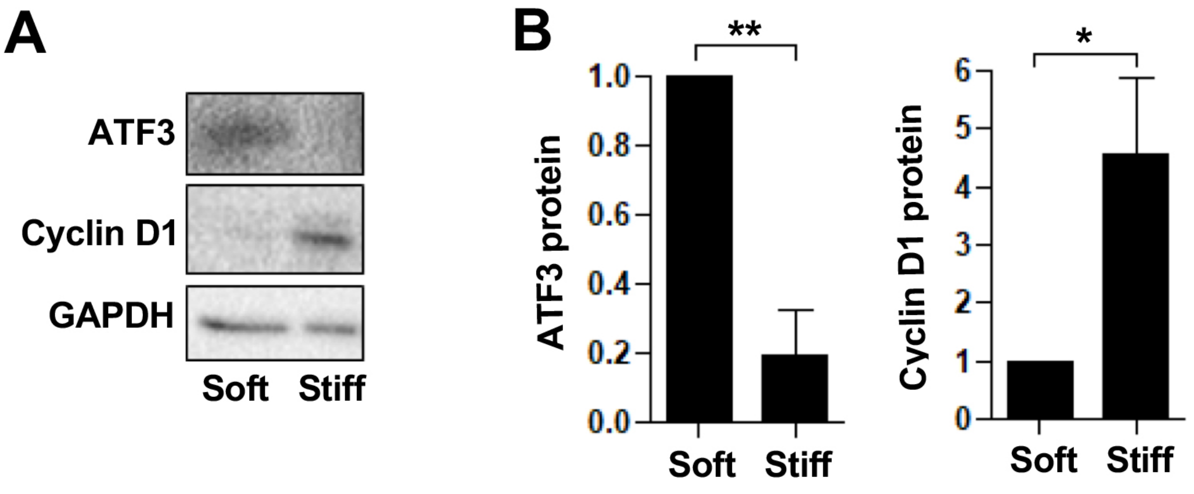

Fig. S3. Inverse effects of ECM stiffness on ATF3 and cyclin D1 protein. Starved MEFs were plated on soft or stiff FN-coated hydrogels with 10% FBS for 24 hr. **(A)** The cells were analyzed by immunoblot for ATF3 and cyclin D1 with GAPDH as the loading control. **(B)** Quantification of immunoblot results in A; the graph shows mean + SD with results normalized to GAPDH abundance and plotted relative to the normalized signal on the soft hydrogels; n=3. Statistical significance was determined by 2-tailed, paired t-tests.

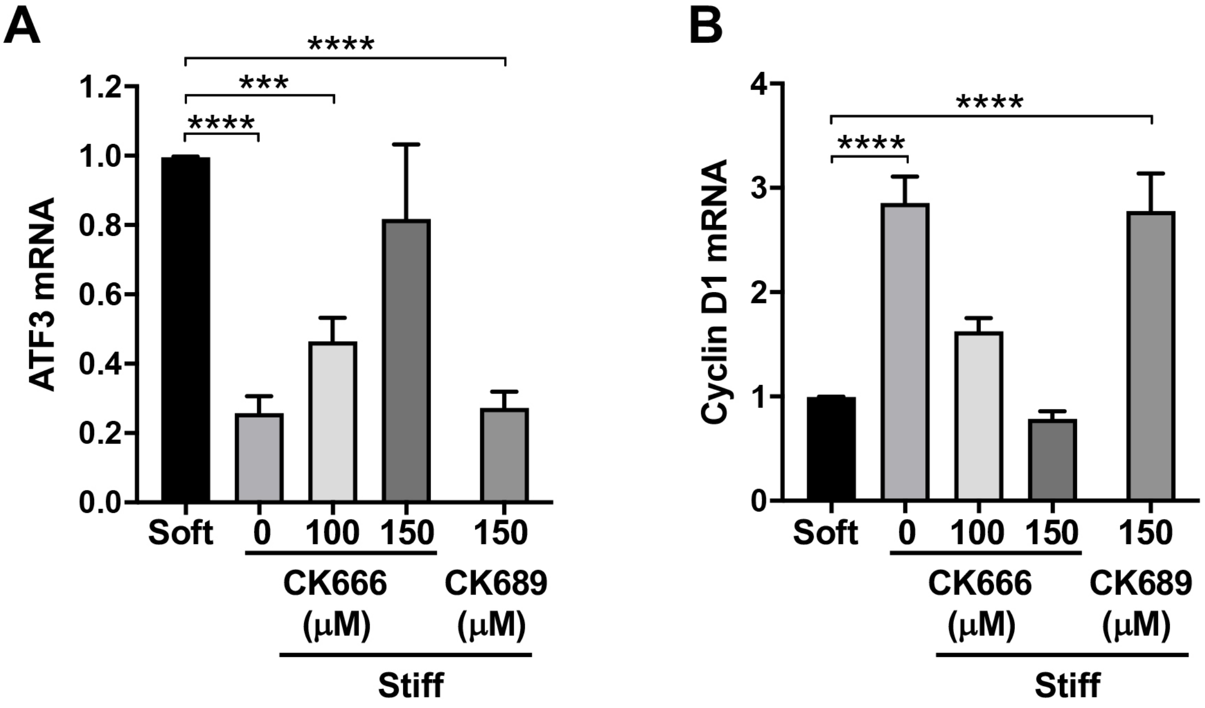

Fig. S4. Involvement of Arp2/3 in the regulation of ATF3 and cyclin D1 mRNAs. Starved MEFs were plated on soft or stiff FN-coated hydrogels with 10% FBS for 9 hr in the presence of DMSO, CK666 (Arp2/3 inhibitor) at 100 μM or 150 μM, or the inactive analog (CK689) at 150 μM. **(A-B)** mRNAs were extracted, and the levels of ATF3 and cyclin D1 mRNA were quantified by RT-qPCR. Results show mean + SE (n=4-6) and are normalized to the soft hydrogels. Statistical significance was determined by one-way ANOVA; asterisks show the results of Dunnett's post-tests performed relative to the soft hydrogel.

Journal of Cell Science • Supplementary information

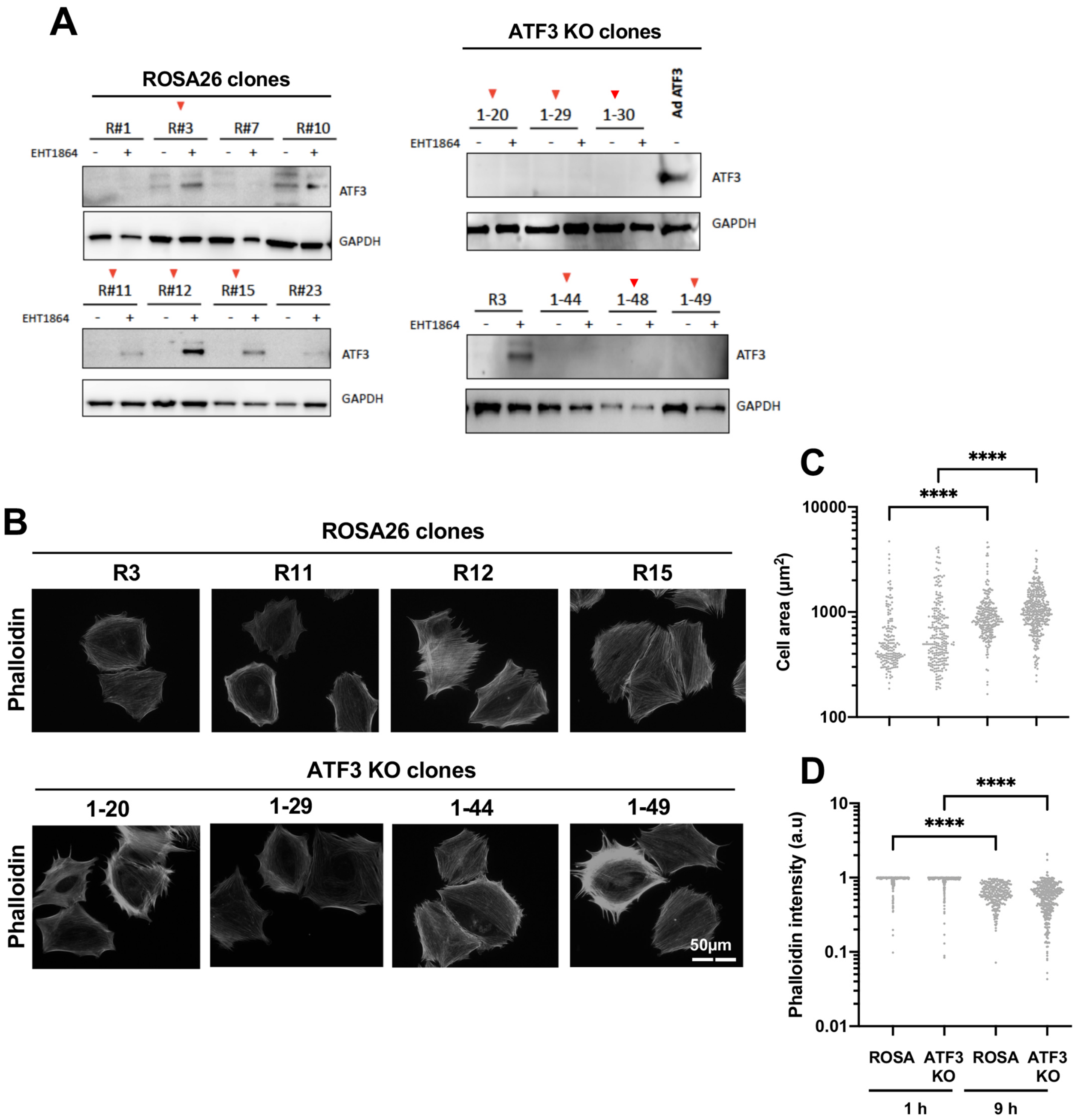

Fig. S5. Identification and characterization of ATF3 KO MEFs. (**A**) Several putative ATF3 deletion clones, including those identified by the ICE Crispr Analysis Tool (Table S2), and a similar number of ROSA26 (control) clones were serum-starved, pre-treated with 10 µM EHT1864 in suspension, and plated on FN-coated tissue culture dishes with 10% FBS-DMEM in the continued presence of EHT1864 for 5 hr before determination of ATF3 protein levels by immunoblot. GAPDH was the loading control. Red arrowheads show several ROSA and ATF3 KO clones with the expected behavior for ATF3. **(B)** Images of control and ATF3 KO MEFs that had been serum-starved and plated on stiff FN-coated hydrogels in DMEM with 10% FBS for 9 hr before fixation and staining with Alexa Fluor™ 594 Phalloidin. **(C-D)** Quantitative analysis of cell area and phalloidin intensity per cell in the ROSA26 controls (R3, R11, R12, R15) and ATF3 KO clones (1-20, 1-29, 1-44, 1-49) as determined using Image J. Graphs show ~170-370 cells analyzed per condition, accrued from 2 independent experiments. Statistical significance is shown for Tukey post-tests of one-way ANOVAs.

Journal of Cell Science • Supplementary information

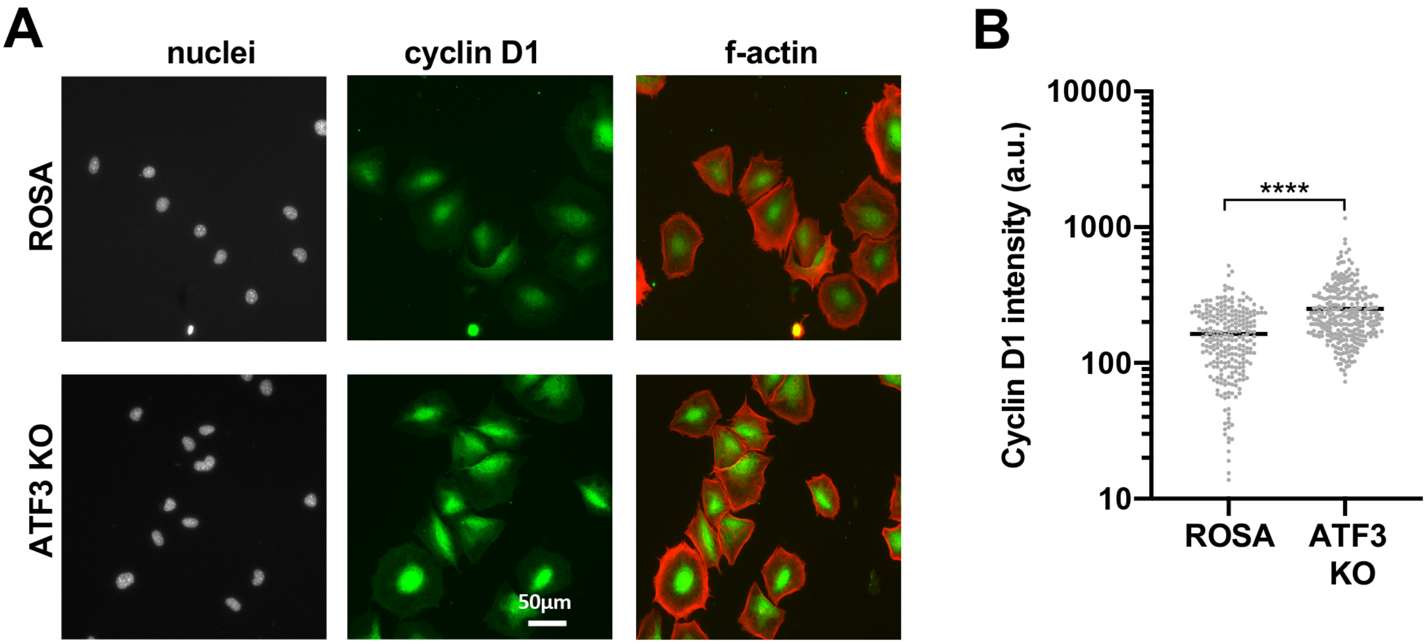

Fig. S6. Nuclear localization of cyclin D1 is not dependent on ATF3. ROSA (control) and ATF3 KO MEFs were serum-starved and plated on stiff FN-coated hydrogels in DMEM-10% FBS for 9 h. **(A)** Representative images of cells stained with DAPI (nuclei), anti-cyclin D1, or phalloidin (f-actin). The panel shows clones ROSA clone R15 and ATF3 KO clone 1-49. **(B)** Cyclin D1 staining intensity quantified from ROSA (control) clones (R3, R11, R12 and R15; n=252) and ATF3 KO clones (1-20, 1-29, 1-44, 1-48, and 1-49; n=308) accrued from 2 independent experiments. The dot plot shows results for each cell and the mean. Statistical significance was determined by a 2-tailed, unpaired t-test.

Uncropped gels for Fig. 1B

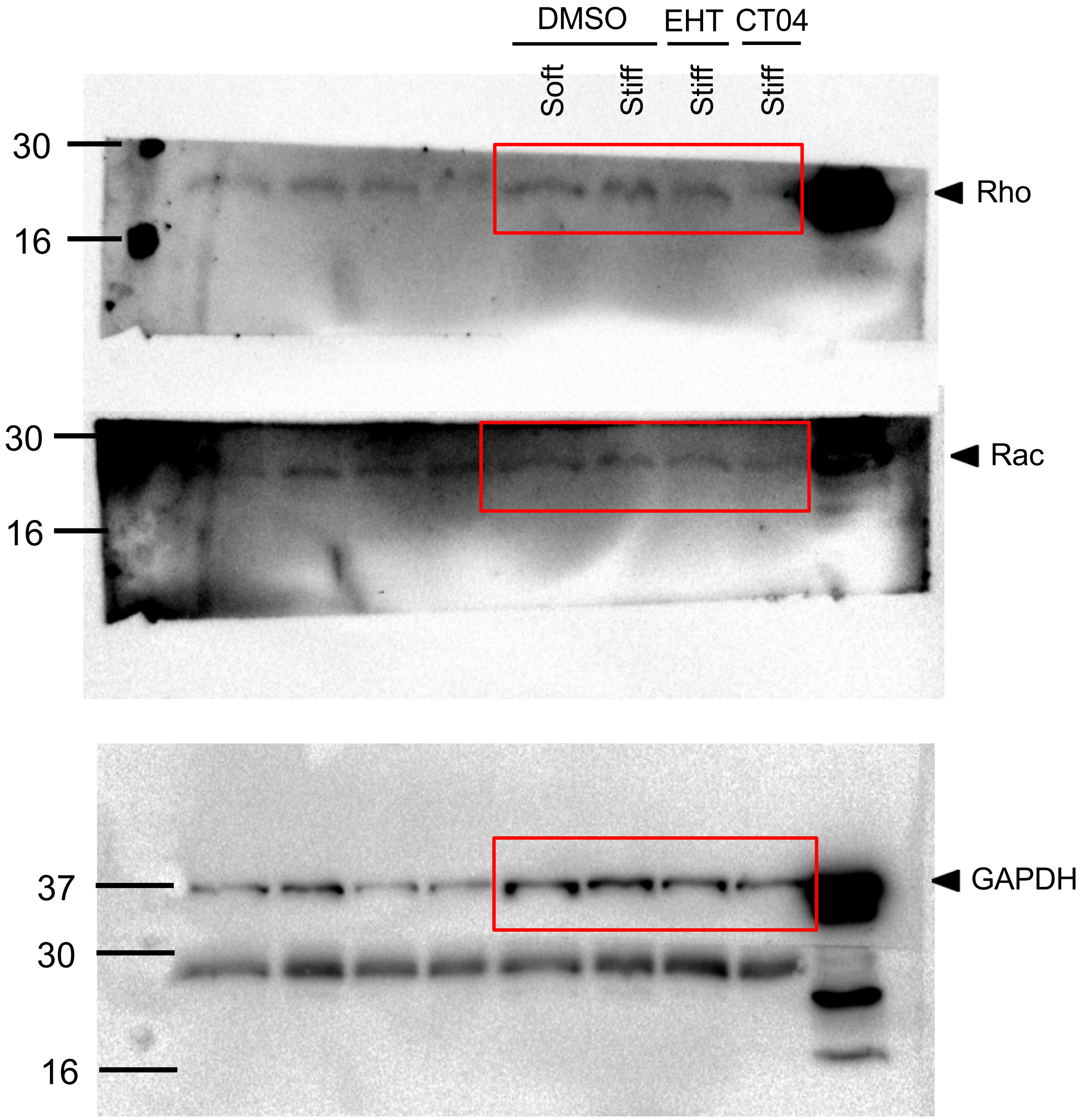

Fig. S7. Blot transparency.

Journal of Cell Science • Supplementary information

Uncropped gels for Fig. 4C

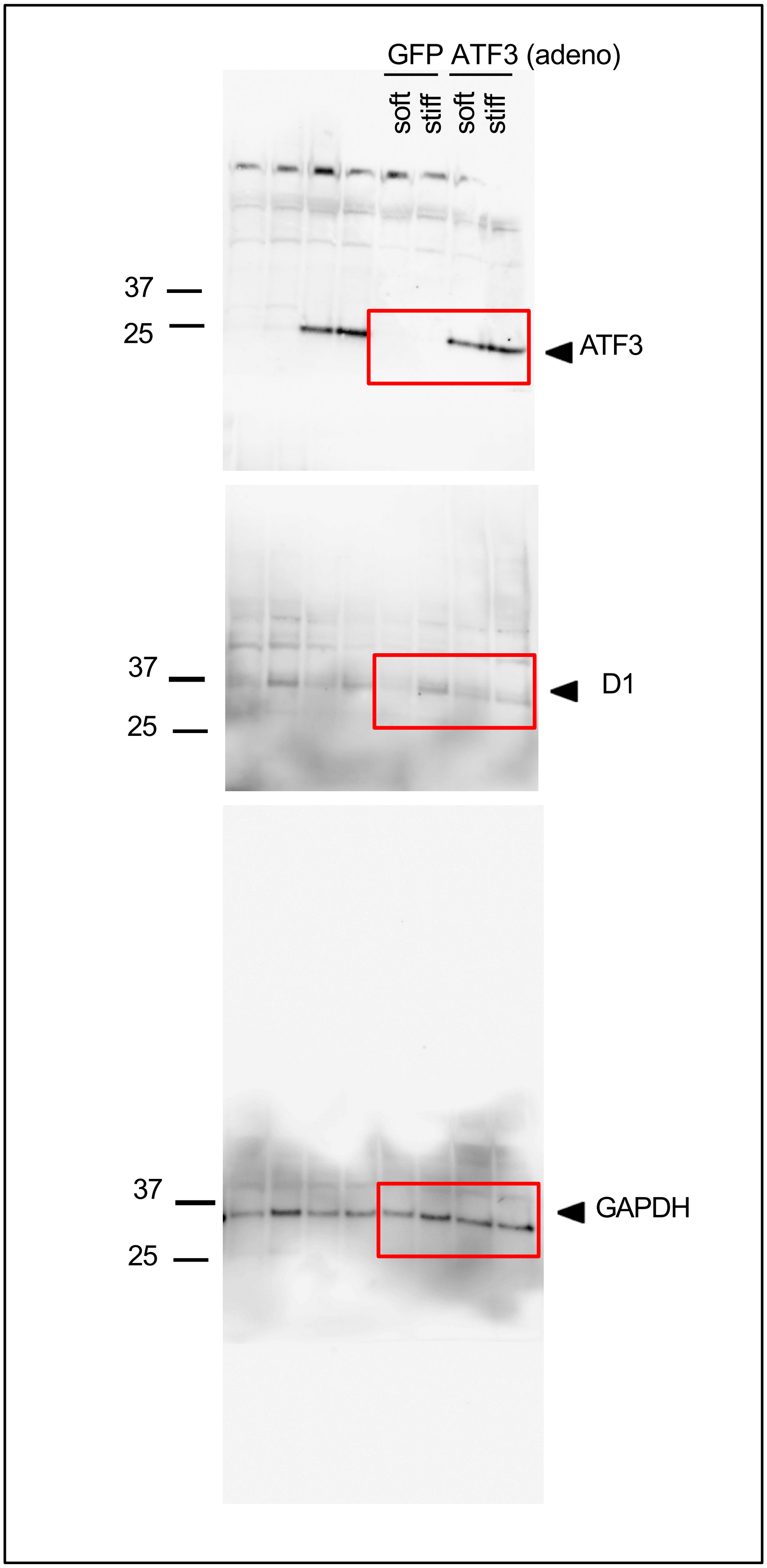

Journal of Cell Science • Supplementary information

Uncropped gels for Fig. 4E

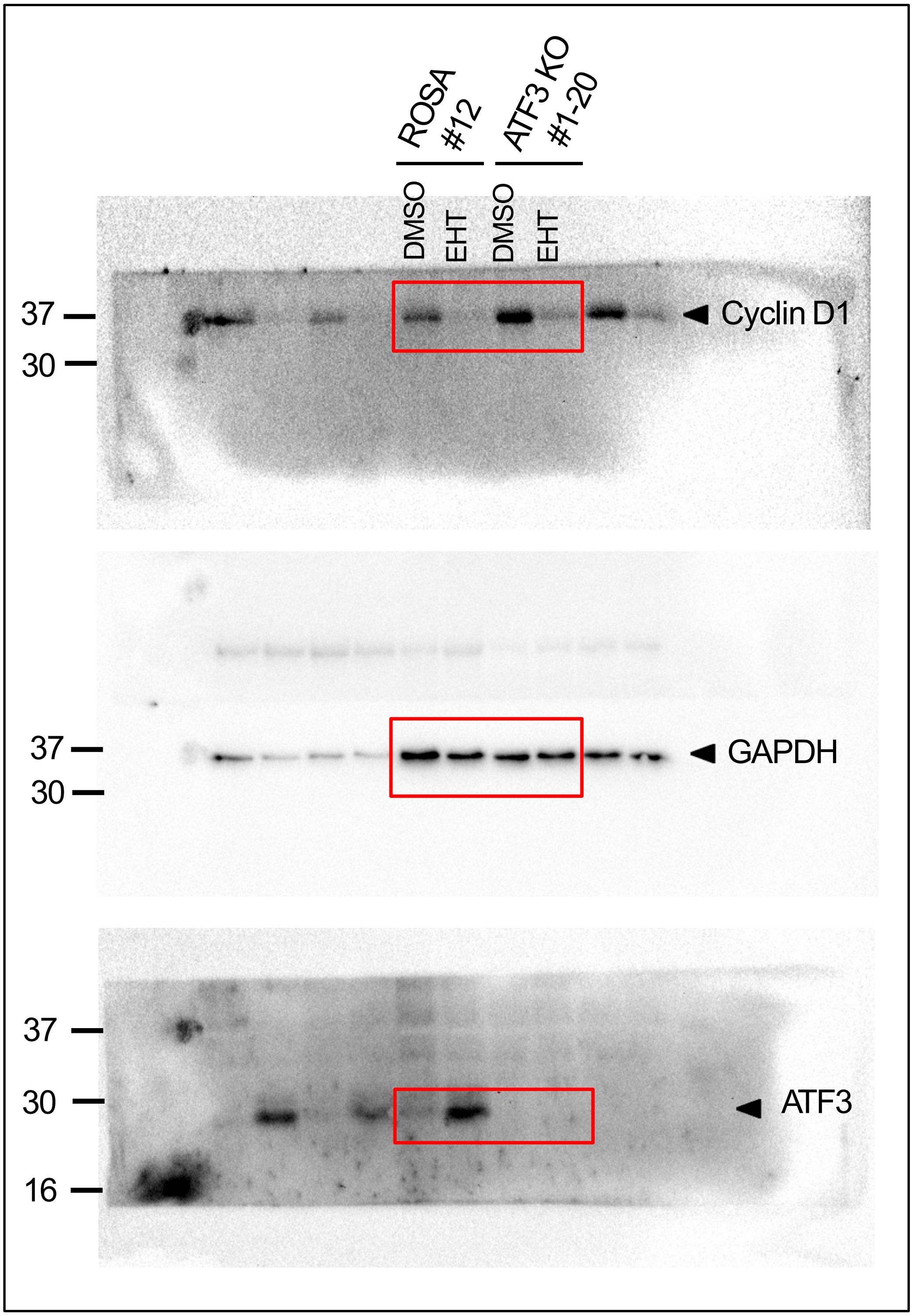

Uncropped gels for Fig. S2

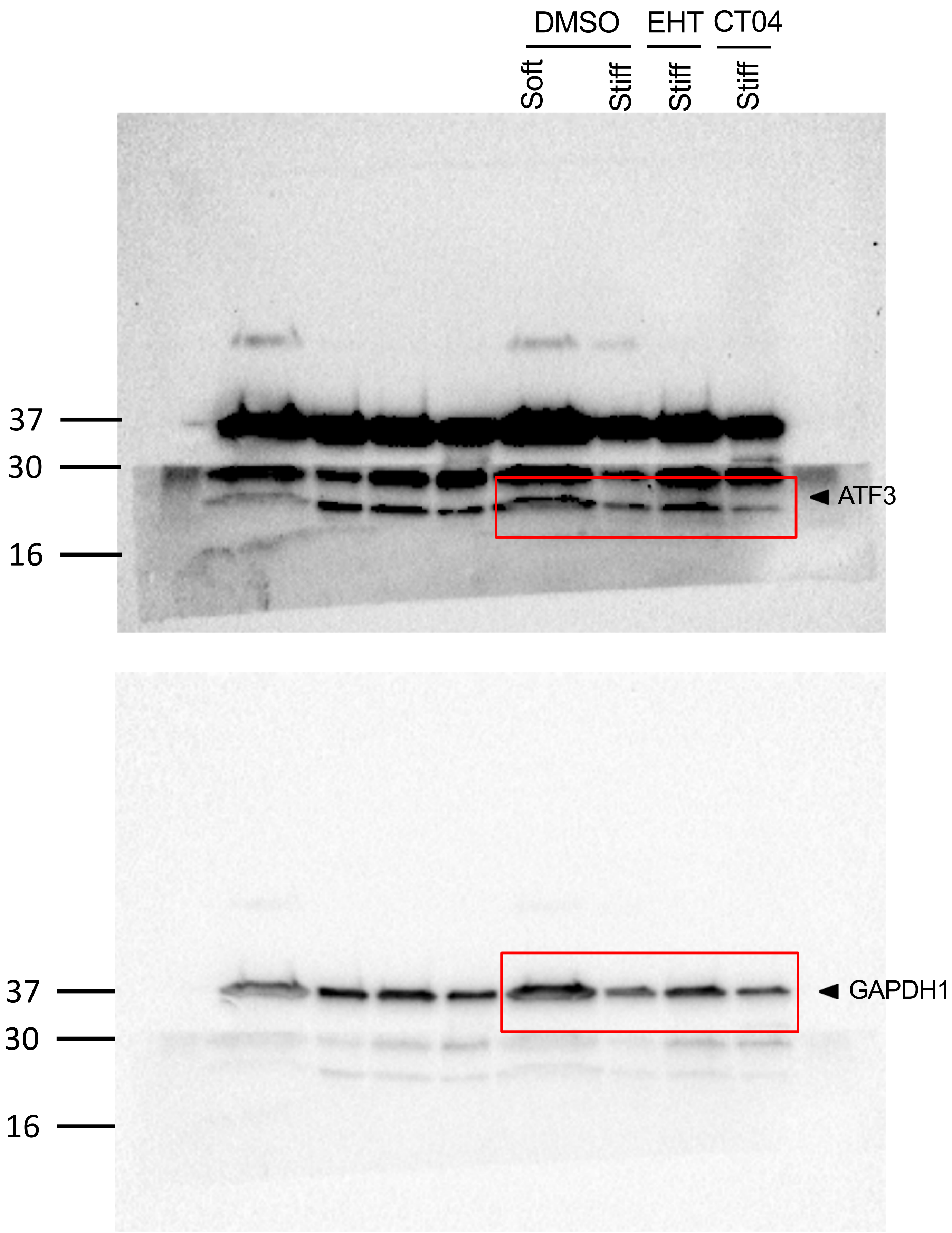

Journal of Cell Science • Supplementary information

Uncropped gels for Fig. S3

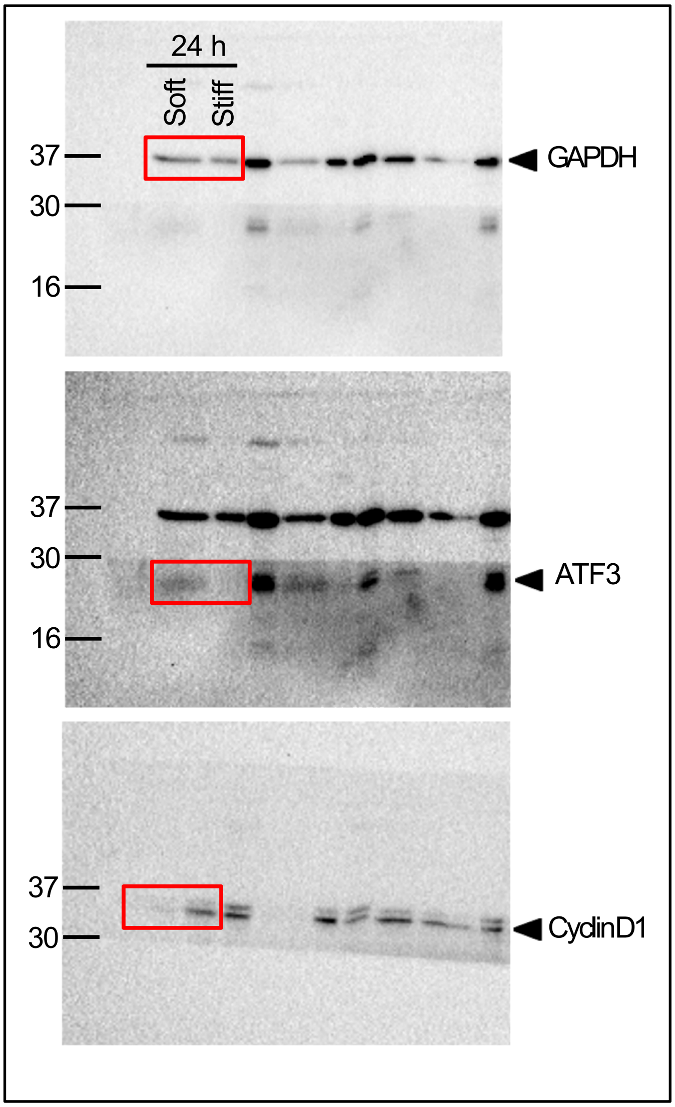

Journal of Cell Science • Supplementary information

Uncropped gels for Fig. S5A

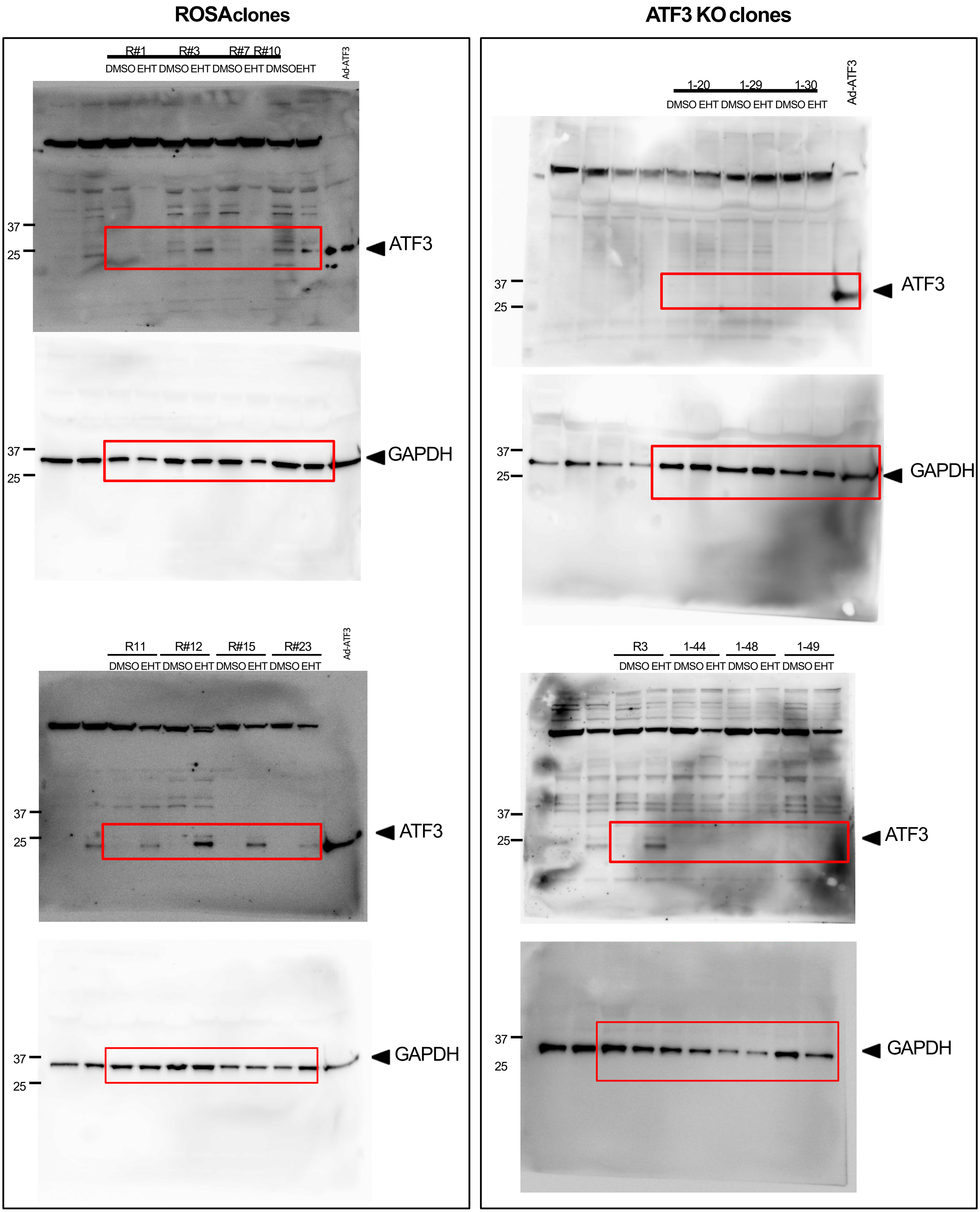

Journal of Cell Science • Supplementary information

Table S1. Gene lists for the Venn diagrams shown in Figure 2

Panel 2A

Stiffness-stimulated and only EHT1864-inhibited (157 genes)

1810013L24Rik
Adamts5
Aebp2
Ahctf1
Ahnak
Akap11
Alg10b
Amer1
Ankhd1
Ankrd17
Ankrd50
Apaf1
Arc
Arhgap35
Arl5a
Atg2b
Birc6
Bmp2k
Bmpr2
Bpnt2
Bptf
Btaf1
Btbd7
Cbfa2t2
Ccnt1
Cdk6
Cert1
Clasp2
Cltc
Cmtm4
Cnot6
Crybg1
Csnk1g1
Dicer1
Dido1
Dip2b
Dmxl1
Dnm3os
Dock5
Dock9
Dr1
Dyrk1a
Elk4
Errfi1
Exoc5
F2r
Fam117b
Fem1b
Fmr1
Garre1
Ggnbp2
Gm14636
Gm37899
Gtf2a1
Hdac4
Hectd1
Kdm5b
Kif13b
Kifap3
Klhl11
Kmt2c
Lats1
Lats2
Lgr4
Lnpep
Lrrc58
Mast4
Mdn1
Med13
Med14
Mical3
Mindy2
Mob1b
Mrtfb
mt-Nd5
Mycbp2
Naa15
Naa50
Nf1
Npat
Nufip2
Nup153
Nup98
Pafah1b1
Pak2
Pank3
Parg
Pdzd8
Peak1
Phc3
Pigm
Ppp1r15b
Prkdc
Psme4
Purb
Rab1a
Ranbp2
Rap1b
Rap2c
Rasa1
Rbbp6
Rc3h2
Ric1
Rictor
Rlf
Rlim
Rmnd5a
Rnf111
Rnf169
Rnf213
Runx1
Sacs
Secisbp2l
Shroom4
Six4
Smg7
Sos1
Sos2
Spred1
Stard9
Sypl
Tanc1
Tasor
Tasor2
Tbc1d8b
Tent4b
Tjp1
Tmem263
Top1
Tpp2
Trib1
Tshz1
Ttc14
Ubr5
Uhmk1
Uhrf1bp1l
Urb2
Usp14
Usp24
Usp9x
Vcpip1
Washc4
Wdr26
Xpo4
Xrn1
Yeats2
Ythdf3
Zbed6
Zbtb1
Zfc3h1
Zfp267
Zfp275
Zfp281
Zfp948
Zfpm2
Zhx3
Zswim6

Stiffness-stimulated and only CT04-inhibited (5 genes)

Ddx58
Exoc8
Irs1
Ptp4a1
Zfp36

Stiffness-stimulated and both EHT1864- and CT04-inhibited (1 gene)

Zfp646

Table S1. Gene lists for the Venn diagrams shown in Figure 2 (continued)

Panel 2B

Stiffness-inhibited and only EHT1864-stimulated (109 genes)

1110038B12Rik
2410006H16Rik
4933434E20Rik
A430005L14Rik
Aimp1
Arfip2
Atf4
Atp6v0b
Atpif1
Bloc1s1
Bola2
Cinp
Cnpy2
Commd1
Cox7a2l
Cst3
Cstf2
Ctsh
Czib
Ddit3
Eef1b2
Erp29
Fau
Ftl1
Gadd45a
Gm10275
Gm11808
Gm12481
Gm14494
Gm15500
Gm2000
Gm26532
Gm4332
Gm6969
Gm7536
Gm8210
Gnptg
Grb2
H1f2
H2ac19
H2bc4
Hoxb2
Ift22
Igbp1
Igfbp2
Jun
Jund
Mri1
Mrpl19
Mrpl21
Mrpl30
Mrpl52
Mt2
Mydgf
Ndufa3
Ndufa6
Ndufa7
Ndufb5
Neurl4
Ost4
Paxx
Pcif1
Rpl11
Rpl12
Rpl13
Rpl14
Rpl15-ps2
Rpl15-ps6
Rpl18
Rpl18a
Rpl21
Rpl22
Rpl23a
Rpl24
Rpl31-ps8
Rpl34
Rpl35
Rpl37
Rpl4
Rpl6
Rpl8
Rps12
Rps13-ps1
Rps17
Rps18
Rps19
Rps20
Rps21
Rps23-ps1
Rps25
Rps27a
Rps27rt
Rps3
Rps5
Rps6
Rps7
Rps7-ps3
Rps8
Rps9
Snhg16
Stoml2
Tmed1
Tmem208
Tmem242
Tpt1-ps3
Uqcc2
Uqcr10
Washc3
Zfas1

Stiffness-inhibited and only CT04-stimulated (5 genes)

Ccdc137
Dusp14
Slc35e4
S100a10
Lsm6

Stiffness-inhibited and both EHT1864- and CT04-stimulated (14 genes)

Atf3*
Dnaja4
Dnajb1
Gm12338
Hspa1a**
Hspa1b
Hspb1
Klf2
Mrps33
Nedd8
Rhob
Rpl31
Rpl36a-ps2
Serf1

*0.84 $\log_2$ fold change for CT04 vs. 2.14 for EHT1864
**not pursued as its calculated adjusted p-value in DESeq2 was undefined

Journal of Cell Science • Supplementary information

Table S1. Gene lists for the Venn diagrams shown in Figure 2 (continued)

Panel 2C
Stiffness-stimulated and EHT1864-inhibited
Transcription factors & co-regulators (23 genes)

Aebp2	Npat	Zbtb1
Btaf1	Nup98	Zfp267
Cbfa2t2	Purb	Zfp275
Cnot6	Rap2c	Zfp281
Dyrk1a	Runx1	Zfp948
Med13	Six4	Zfpm2
Med14	Tshz1	Zhx
Mrtfb	Zbed6	

Histone modifications (6 genes)
Fmr1
Ubr5
Parg
Yeats2
Naa50
Dr1

Histone modifications and Transcription factors and co-regulators (5 genes)
Elk4
Hdac4
Kdm5b
Kmt2c
Rlf

Panel 2D
Stiffness-inhibited and EHT1864-stimulated
Transcription factors & coregulators (9 genes)
Atf3
Atf4
Ddit3
Dnajb1
Hoxb2
Hspa1a (see note for panel 2B)
Jun
Jund
Klf2

Histone modifications (1 gene)
H1f2

Journal of Cell Science • Supplementary information

Table S1. Gene lists for the Venn diagrams shown in Figure 2 (continued)

Panels E-F
Gene list, fold changes (FC) and adjusted p-values (padj) for the graphs shown in Figures 2E-F.

Gene	*FC stiff/soft*	*padj stiff/soft*	*FC stiff ± EHT*	*padj stiff ± EHT*
Aebp2	0.362360035	0.039872997	-0.430363934	0.003114619
Atf3	-0.842902142	1.24811E-11	2.145058258	1.93545E-98
Atf4	-0.37550875	0.00280697	1.094335394	1.56274E-34
Btaf1	0.464672293	0.008971385	-0.324555895	0.04343359
Cbfa2t2	0.41488484	0.011717977	-0.339958504	0.018569795
Cnot6	0.503573055	0.013761436	-0.447533286	0.011382425
Ddit3	-0.455580575	0.017797817	1.232648422	2.23548E-19
Dnajb1	-0.823217294	3.24098E-10	3.039256505	2.3813E-185
Dr1	0.478122321	0.013496317	-0.416349259	0.01323619
Dyrk1a	0.40944526	0.004902845	-0.42730384	0.000374685
Elk4	0.441802107	0.008318563	-0.518755581	0.000141077
Fmr1	0.64383477	0.002564592	-0.414247267	0.032879518
H1f2	-0.599990161	0.010656195	0.965007028	7.64479E-08
Hdac4	0.357327008	0.016954218	-0.582158879	5.14256E-07
Hoxb2	-0.396567327	0.014372579	0.437993522	0.001075301
Jun	-0.537149245	0.005858218	1.801131885	6.29779E-40
Jund	-0.664710836	3.55088E-07	0.881502801	2.63829E-16
Kdm5b	0.394928769	0.009067506	-0.444544135	0.000317503
Klf2	-1.32456878	7.17855E-14	1.096519866	8.85946E-12
Kmt2c	0.605275299	0.041782327	-0.595477572	0.019929676
Med13	0.937533168	0.001556194	-0.584996931	0.030493793
Med14	0.425412702	0.04245209	-0.52651539	0.002082577
Mrtfb	0.47663481	0.013024444	-0.414162701	0.012859084
Naa50	0.654766699	5.21957E-05	-0.395677938	0.007332563
Npat	0.423277831	0.018719279	-0.422098456	0.005748077
Nup98	0.441426877	0.017037315	-0.328370145	0.049235075
Parg	0.45320743	0.027580408	-0.410129915	0.022023721
Purb	0.851574605	0.001415271	-0.73580509	0.001118295
Rap2c	0.52841838	0.000446182	-0.432019665	0.000695973
Rlf	0.41422215	0.006242845	-0.636060685	5.16445E-08
Runx1	0.692994542	0.001947058	-0.952609268	5.12789E-08
Six4	0.628202539	0.002009872	-0.998712042	1.47086E-10
Tshz1	0.445936334	0.039202763	-0.556967227	0.001604112
Ubr5	0.538590616	0.022150642	-0.459121996	0.026967911
Yeats2	0.408365022	0.012058095	-0.389266858	0.004684534
Zbed6	0.714189321	0.030122649	-0.723758197	0.00948818
Zbtb1	0.456335088	0.004845731	-0.70166333	2.05241E-08
Zfp267	0.354369365	0.021576453	-0.391180118	0.002202342
Zfp275	0.414505347	0.001832134	-0.450659831	3.11125E-05
Zfp281	0.448880655	0.022560895	-0.94108134	1.93456E-10
Zfp948	0.715492521	0.002439758	-0.628019391	0.001681668
Zfpm2	0.506331146	0.029174177	-0.524693627	0.00734088
Zhx3	0.511305749	0.000295488	-0.381625136	0.001734441

Table S2. Identification of putative ATF3-depleted MEF clones. MEF clones from the Crispr transfection were lysed in QuickExtract™ DNA Extraction buffer (Lucigen). Genomic DNA was extracted by two cycles of vortexing (15 sec) and heating (first at 68°C for 15 min and then at 95°C for 10 min). The DNA was subjected to PCR amplification, the purified PCR products were sequenced using a reverse ATF3 primer for gRNA-1 (GGTGCACACTATACCTGCTC), and the sequences were analyzed using the ICE CRISPR Analysis Tool (Synthego). A Knockout (KO) Score was generated for each clone. The KO score represents the proportion of cells that have either a frameshift or 21+ bp indel. We selected several clones with KO scores >90 for further analysis. Clone 1-5 was not used in this study.

Clone ID	KO-Score
1-5	95
1-20	94
1-29	94
1-48	94
1-44	94
1-49	95

Journal of Cell Science • Supplementary information

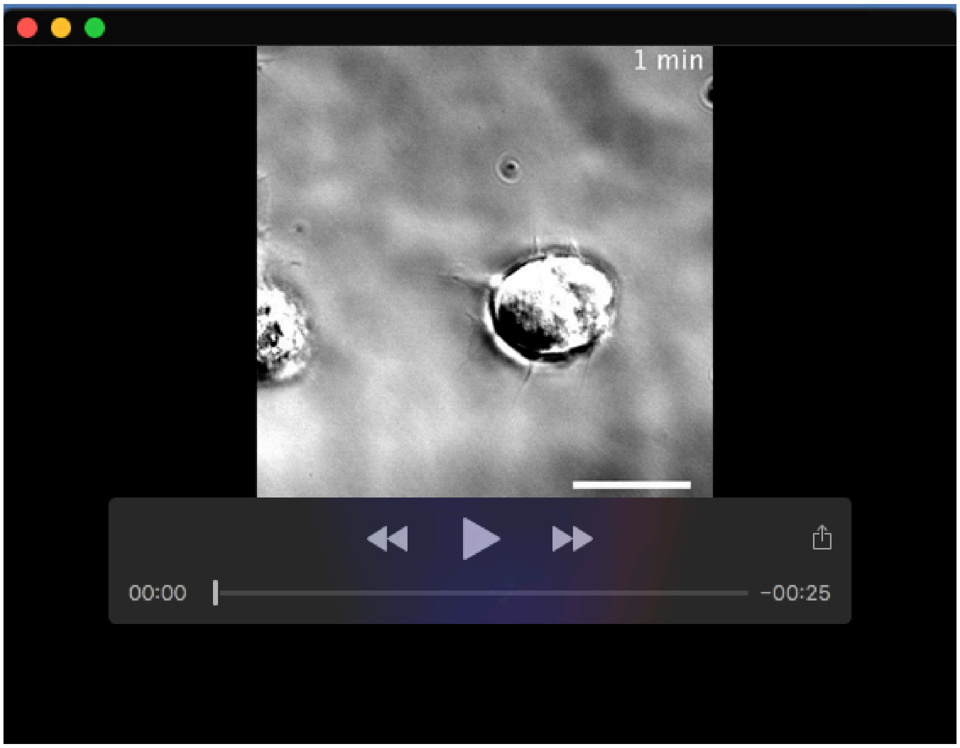

Movie 1. Spreading of control MEFs on stiff hydrogels. MEFs were cultured in DMEM-10% FBS to 70-80% confluency, trypsinized, and pre-incubated in suspension for 30 min in DMEM with 1 mg/ml BSA and vehicle (DMSO) as described in Materials and Methods. The cells were then plated on FN-coated stiff hydrogels in DMEM-10% FBS in the continued presence of DMSO. The cells were imaged every 15 sec for 15 min using the 40X objective of a Zeiss Axio Observer 7 inverted microscope. Scale bar = 30 μm. Movies were selected to show typical responses; n~100 cells accrued from 2 independent experiments.

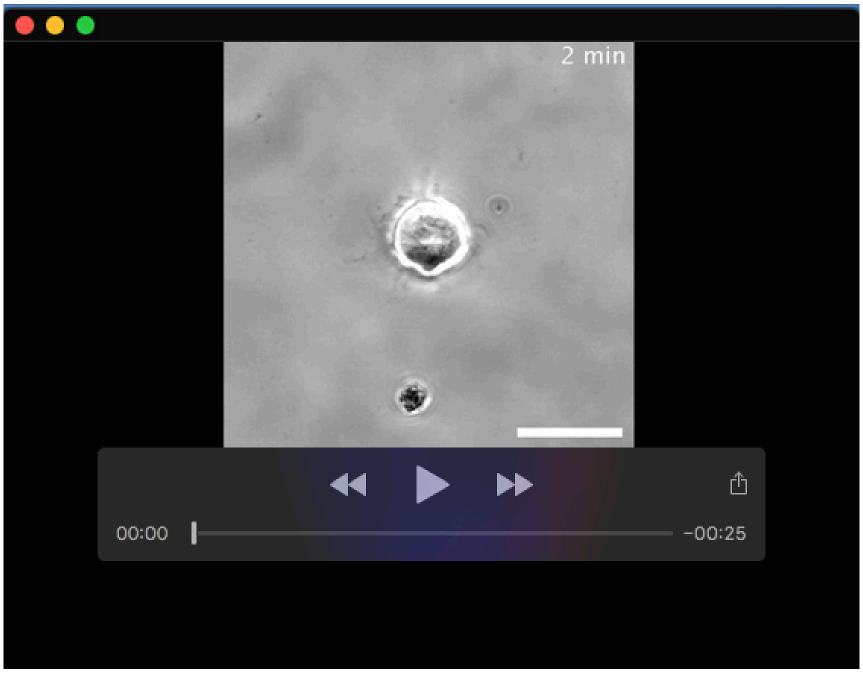

Movie 2. Spreading of EHT1864-treated MEFs on stiff hydrogels. MEFs were cultured as for Movie 1 but treated with EHT1864. Scale bar = 30 µm.

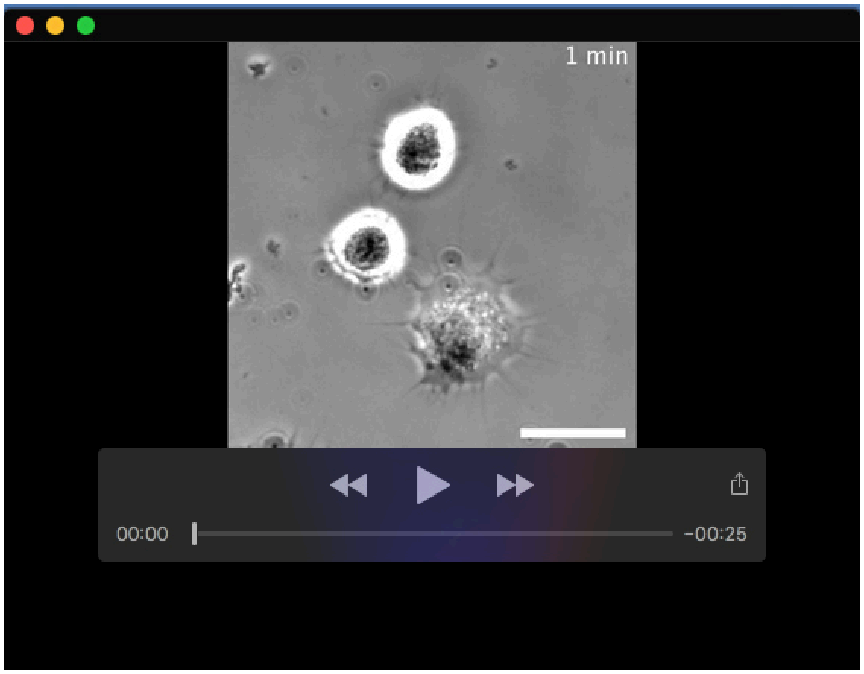

Movie 3. Spreading of CT04-treated MEFs on stiff hydrogels. MEFs were cultured as for Movie 1 but treated with CT04. Scale bar = 30 µm.

Journal of Cell Science • Supplementary information
